# Study of Optimal Perimetric Testing In Children (OPTIC): developing consensus and setting research priorities for perimetry in the management of children with glaucoma

**DOI:** 10.1038/s41433-021-01584-0

**Published:** 2021-06-21

**Authors:** Dipesh E. Patel, Phillippa M. Cumberland, Bronwen C. Walters, Joseph Abbott, John Brookes, Beth Edmunds, Peng Tee Khaw, Ian Christopher Lloyd, Maria Papadopoulos, Velota Sung, Mario Cortina-Borja, Jugnoo S. Rahi, For the OPTIC Study Group, Peng Tee Khaw, Bronwen Walters, Phillippa Cumberland, Isabelle Russell-Eggitt, Christine Timms, John Brookes, Anthony Moore, Maria Papadopoulos, David Garway-Heath, Ananth Viswanathan, Alki Liasis, David Crabb, Mario Cortina-Borja, Dipesh Patel, Jugnoo Rahi

**Affiliations:** 1 Life Course Epidemiology and Biostatistics Section, UCL GOS Institute of Child Health, London, UK; 2 Ulverscroft Vision Research Group, London, UK; 3 NIHR Biomedical Research Centre at Moorfields Eye Hospital NHS Foundation Trust and UCL Institute of Ophthalmology, London, UK; 4 Great Ormond Street Hospital for Children NHS Foundation Trust, London, UK; 5 Department of Ophthalmology, Birmingham Children’s Hospital, Birmingham, UK; 6 Birmingham Institute for Glaucoma Research, Institute of Translational Medicine, Birmingham, UK; 7 Glaucoma Service, Moorfields Eye Hospital NHS Foundation Trust, London, UK; 8 Department of Ophthalmology, Casey Eye Institute, Oregon Health & Science University, Portland, OR USA; 9 Manchester Royal Eye Hospital, Central Manchester University Hospitals NHS Foundation Trust, Manchester, UK; 10 Birmingham and Midland Eye Centre, Sandwell and West Birmingham Hospitals NHS Trust, Birmingham, UK; 11 Clinical Epidemiology, Nutrition and Biostatistics Section, UCL GOS Institute of Child Health, London, UK; 12 Moorfields Eye Hospital NHS Foundation Trust, London, UK; 13 UCL Institute of Ophthalmology, London, UK; 14 City University, London, UK

**Keywords:** Paediatrics, Glaucoma

## Abstract

**Background:**

Perimetry is important in the management of children with glaucoma, but there is limited evidence-based guidance on its use. We report an expert consensus-based study to update guidance and identify areas requiring further research.

**Methods:**

Experts were invited to participate in a modified Delphi consensus process. Panel selection was based on clinical experience of managing children with glaucoma and UK-based training to minimise diversity of view due to healthcare setting. Questionnaires were delivered electronically, and analysed to establish ‘agreement’. Divergence of opinions was investigated and resolved where possible through further iterations.

**Results:**

7/9 experts invited agreed to participate. Consensus (≥5/7 (71%) in agreement) was achieved for 21/26 (80.8%) items in 2 rounds, generating recommendations to start perimetry from approximately 7 years of age (IQR: 6.75–7.25), and use qualitative methods in conjunction with automated reliability indices to assess test quality. There was a lack of agreement about defining progressive visual field (VF) loss and methods for implementing perimetry longitudinally.

Panel members highlighted the importance of informing decisions based upon individual circumstances—from gauging maturity/capability when selecting tests and interpreting outcomes, to accounting for specific clinical features (e.g. poor IOP control and/or suspected progressive VF loss) when making decisions about frequency of testing.

**Conclusions:**

There is commonality of expert views in relation to implementing perimetry and interpreting test quality in the management of children with glaucoma. However, there remains a lack of agreement about defining progressive VF loss, and utilising perimetry over an individuals’ lifetime, highlighting the need for further research.

## Introduction

Childhood glaucoma is a rare, potentially blinding optic neuropathy characterised by raised intraocular pressure (IOP) and optic nerve damage [1]. Five in 100,000 children born in Great Britain each year are diagnosed with the most frequent form of childhood glaucoma, primary congenital glaucoma [2, 3], which is usually primarily managed surgically, with further surgery and/or topical treatment required to mitigate disease progression [4].

Monitoring children with glaucoma presents different challenges to the management of adults in whom measurement of IOP, assessment of optic disc morphology and visual field (VF) assessment together form a core triad of the clinical assessment of progression [5, 6], with an emerging role for imaging techniques [7, 8]. IOP measurement and direct visualisation of the optic disc are both more challenging but nevertheless achievable with appropriate approaches in children [1]. Optimal approaches to assessing the VF are less well defined. To address this, we have previously investigated the relative strengths of static, kinetic, and combined static and kinetic techniques in children with glaucoma [9], adding information about the use of optimised static perimetry algorithms such as the Humphrey SITA 24-2 FAST (Swedish Interactive Thresholding Algorithm, Carl Zeiss Meditec AG, Jena, Germany) and Octopus G-TOP (Tendency-Oriented Perimetry, Haag-Streit Holding AG, Koeniz, Switzerland) to existing data on rarebit [10], supra-threshold [11] and game-based techniques [12]. Our research also demonstrated the utility of combined static/kinetic perimetry for monitoring VF defects in children with severe glaucoma [9]. However, there remains a dearth of information about how these data should be translated into improving clinical decision-making in long-term care.

To address the challenge of translating current research findings into practice, we undertook an expert consensus development study with an invited expert panel [13, 14] to extend previous expert consensus guidance [1] that advocates perimetry, but provides limited information on approach or how findings should affect management decisions. Where a divergence of opinions remains, we suggest areas requiring further research.

## Methods

Consultant ophthalmologists with specialist expertise in paediatric glaucoma were identified through membership of the UK Paediatric Glaucoma Society (an affiliated society of the International Glaucoma Association), and invited to participate in a modified Delphi consensus process [14]. Prospective panel members were also asked to nominate other experts within the field. Panel eligibility was limited to consultants with experience of managing children with glaucoma and UK-based training. Panel members thus had shared experience of and access to similar perimetric technology.

Panel members were asked to complete, without conferring, questionnaires delivered electronically (PDF forms created in LibreOffice Writer v5.3.6.1, ©The Document Foundation). The first-round questionnaire encompassed all aspects of undertaking and applying perimetry alongside a free text comment section (online supplementary material). Questions either asked for specific values (e.g., age at which perimetry should be started), agreement with a statement (5-point Likert scale, from strongly disagree to strongly agree), or multiple-choice responses (i.e. presence of mild, moderate or severe VF loss).

The consensus process comprises iterations in which agreement is re-examined using responses from previous rounds until a pre-defined threshold of agreement has been achieved [15]. Responses were tabulated and analysed in Microsoft Excel^®^. In addition, any new areas identified by panel members are also explored. At the end of the consensus process, statements were formulated into proposed recommendations for practice by the first author (DEP). The panel was asked to review these and agree or disagree whether these accurately reflected the consensus statements from which they were derived. This study did not require institutional ethics board approval. Consent was not required as this was evidence synthesis through a Delphi process by a research group rather than primary research on participants.

## Results

Seven of nine (78%) invited experts participated. Panel members (all of whom are also co-authors) are listed in the acknowledgements.

Agreement during consensus development was defined as 5 or more of 7 experts (i.e. ≥70%) in agreement. The questionnaires were administered between November 2017 and February 2018.

Two rounds were required to achieve the consensus reported here. A third round was offered to the panel, focussing on areas where agreement was not achieved in previous rounds. However, the panel felt that, due to a lack of evidence on which to base further discussions, it was unlikely that further iterations would result in a greater consensus. Agreement with the verbatim statements about aspects of perimetry is shown in Table 1.

**Table 1:** Verbatim consensus statements with associated panel agreement.

Full agreement (7/7)	**Minimum age for starting perimetry**
	Combined static and kinetic perimetry can be started in children from 7.75 years of age (IQR: 7.5–9.5)
	**Assessing perimetric test quality**
	False positives are a useful measure of test quality
	False positive values over 15% (IQR: 12.5–20) indicate a test of poor quality^a^
	False negative values over 20% (IQR: 12.5–22.5) indicate a test of poor quality
	Fixation losses are susceptible to artefact (such as head movement and incorrect initial plotting of the blind spot)
	Assessing patient behaviour qualitatively (documenting co-operation, response to stimuli, fixation, and behaviour etc.) is useful for assessing perimetric test quality
	Qualitative (examiner) comments about test quality should always be used in adjunct to quantitative measures
	**Test selection**
	In children, due to poor concentration, shorter algorithms are preferable to their longer counterparts
	Shorter algorithms are useful to train children before undertaking longer algorithms
Good agreement (5/7 or 6/7)	**Minimum age for starting perimetry**
	Simple static or kinetic perimetry should be started from approximately 7 years of age (IQR: 6.75–7.25)
	**Assessing perimetric test quality**
	False negatives are a useful measure of test quality
	Fixation losses are a useful measure of test quality
	Fixation loss values over 15% (IQR: 10–22.5) indicate a test of poor quality
	**Test selection**
	Selecting a smaller test area (24°) can offer a compromise of ease, practicality, patient fatigue and information
	The presence of moderate/severe VF loss is an indication to quantify VF extent using kinetic perimetry
	Kinetic perimetry can be a useful adjunct to static testing in those with co-operation too poor for short static testing
	Combining static perimetry and assessment of the far-peripheral field using kinetic perimetry is useful in assessing visual fields in children with glaucoma
	**Use of perimetry in routine clinical practice**
	Fellow eyes in unilateral glaucoma can serve as ‘controls’ within individual children, aiding monitoring of visual field progression
	Perimetry in children should be undertaken routinely every 7.5 months (IQR: 6–11.25)
	More frequent testing is warranted if there is suspicion of VF deterioration or poor IOP control
	Ideally, children should be assessed with the same perimeter/algorithm throughout childhood
No agreement (<5/7)	**Test selection**
	Assessing an area of 30° is recommended
	Assessing an area of 24° is recommended
	**Assessing progression**
	Evidence of VF progression is defined as: Loss of 2 dB (IQR: 2–2.375) mean deviation (MD) using data from at least 3 (IQR: 2.5–3) consecutive tests.
	**Use of perimetry in routine clinical practice**
	Longer algorithms (i.e., SITA Standard vs. FAST) offer greater precision in detecting progressive VF loss
	If using shorter algorithms early in childhood (e.g., SITA FAST and G-TOP), children/young people should be switched to longer algorithms (e.g., SITA Standard and G) when appropriate

^a^Missing values for one respondent.

Panel members commonly reported that the choice of when to start perimetry is affected by the individual child’s ability to comply with testing and that suggested values, such as frequency of testing or test selection, are affected by the individual’s clinical condition. Caution was recommended when interpreting values generated by automated reliability indices. One panel member stated that thresholds for interpreting automated indices depends on the algorithm used. In general, respondents were keen to emphasise that recommendations should be tempered by the clinical context of individual cases, and that generated guidance should reflect these views. No panel member raised the possibility of using game-based assessments (though these may become a feature of future practice as innovations are commercialised) or confrontation VFs.

The proposed recommendations (generated from consensus statements in Table 1 and approved by the panel) are shown in Table 2. These incorporate areas of both agreement and disagreement across all five topic areas. The panel generated a definition for progressive VF loss in the first round, but did not find this definition to be suitable when re-assessed in the second-round questionnaire.

**Table 2:** Agreed consensus recommendations for perimetry in childhood glaucoma.

Consensus recommendations
**Minimum age for starting perimetry**
Start simple static or kinetic perimetry from approximately 7 years of age (IQR: 6.75–7.25)
Start combined static and kinetic perimetry from 7.75 years of age (IQR: 7.5–9.5)
**Assessing perimetric test quality**
Automated measures of false positives, false negatives and fixation losses are useful in interpreting test quality
Poor quality is indicated by:
False-positive values over 15% (IQR: 12.5–20)
False-negative values over 20% (IQR: 12.5–22.5)
Fixation loss values over 15%, though these are susceptible to artefact
Patient behaviour should be assessed qualitatively (by examiner) and results always used in adjunct to quantitative measures
**Test selection**
*Static*
Use shorter algorithms (e.g., SITA FAST rather than standard)
Shorter algorithms are useful to train children before undertaking longer tests
Use either a 30 or 24° test area, selecting a smaller area (24°) if necessary to improve the likelihood of capturing useful information
*Kinetic*
In children with moderate/severe VF loss, quantify VF extent using kinetic perimetry
If co-operation with static perimetry is likely to be poor, attempt kinetic-only perimetry
*Combined static/kinetic*
Use combined perimetry where possible
**Assessing progression**
No fixed definition of progressive VF loss exists
**Use of perimetry in routine clinical practice**
When interpreting results in children with unilateral glaucoma, to aid monitoring of visual field progression, use fellow eyes as ‘controls‘
Assess fields routinely every 7.5 months (IQR: 6–11.25)
If there is suspicion of VF deterioration or poor IOP control, assess VFs more frequently
Assess with the same perimeter/algorithm throughout childhood

## Discussion

To our knowledge, this is the first study since the publication of World Glaucoma Association (WGA) consensus guidelines in 2013 [1] (and thus incorporating information from new perimetric research) to investigate expert opinion on the use of perimetry in the management of children with glaucoma. We report here strong agreement (≥6/7 (≥85%) panel members) for 16/26 (61.5%) items, demonstrating almost uniform agreement with regards to when to start perimetry and the need to account for quantitative and qualitative reports of test quality when interpreting outcomes. Strong agreement also existed about static techniques, but there was less strong agreement in relation to the use, and role, of kinetic perimetry—a less commonly used technique. Thus, the guidance generated here builds on WGA guidelines, adding information about interpreting test quality, static algorithm selection, test area and monitoring practices. In particular, this new guidance highlights the value and use of kinetic perimetry, recommending the use of a combined static/kinetic approach where possible.

Areas of disagreement (<70% panel members in agreement) likely reflect the paucity of evidence relating to the use of perimetry in long-term management. For example, there is disagreement about the value of, and need to, switch to longer algorithms later in childhood, which contrasts with current guidance [1]. Panel members here placed greater emphasis on consistency of testing over time using algorithms that have been shown to have equivalent precision for detecting progression in adults [16], potentially allowing better comparability across an individuals’ lifetime. Similarly, choices about test area highlight the difficulties clinicians face, and the compromise often required between test area (i.e. information), duration (i.e. fatigue from detailed algorithms) and reliability.

Panel selection, composition and size are important potential sources of bias in consensus studies [14]. The decision to limit panel eligibility was based on the standard model of care delivery in the UK, in which consultants are responsible for final management decisions e.g. the need for further intervention in the presence of suspected or known disease progression. Thus, wider professionals, whilst able to provide diverse views about undertaking perimetry, would not necessarily improve the ability to provide guidance relating to the role of perimetric test outputs in management decisions. Childhood glaucomas have particular age-dependent characteristics such as concurrent amblyopia, reversibility of disc cupping and glaucomatous globe enlargement, which are less familiar to the surgeon whose practice is exclusively concerned with adult disease. Thus, specialists who work exclusively with adults with glaucoma were not eligible, as the clinical expertise required to manage these cases is not transferable to children.

Asking panel members to recommend other eligible candidates risks bias, in which only those with similar views are nominated to the panel [14]. However, given the small number of eligible members, this is unlikely to have occurred here. The ‘ideal’ Delphi panel size is disputed and suggestions vary between 6 and 11 [17], 10 and 30 or hundreds [18] of people. We believe this panel is representative of expertise in UK paediatric glaucoma services. Internationally, methods for assessing VFs in children vary widely, as do available perimeters, affecting the generalisability of the findings we report to those with access to technology as described here.

The consensus was achieved in two rounds, which likely reflects the small number of panel members and similarities in practice across UK-trained ophthalmologists, as well as the limited number of questionnaire items due to the chosen narrow focus on perimetry in a single rare disease. The use of a modified Delphi approach, rather than a nominal group technique, allowed each individual member to contribute equally to the process and enabled participation from multiple geographic locations, but the lack of face-to-face interviews or group discussions precludes our ability to comment in-depth about whether consensus could have been achieved on items after deeper discussions/debate in person. It is likely that our chosen format also affected the ability to generate new ideas, though the impact of this is limited, as this exercise was primarily designed to interrogate known unknowns regarding existing, commonly used approaches. Nevertheless, using available comment sections, panel members generated six new items from the first round, which were included in the second-round questionnaire.

Previous consensus work [1] predates recently published literature, but similarities with this current study exist in relation to the recommended age at which to start perimetry. Our consensus recommendations, as shown in Table 2, also add new information about interpreting test quality, and provide detailed information regarding the relative strengths and use of commonly available perimetric approaches, which compares well to extant literature [9].

Whilst surgical innovations are leading clinicians to achieve effective IOP control with fewer procedures in children [19], the paucity of robust longitudinal research into perimetry limits the ability to use perimetric data for clinical trial endpoints in children and uncertainty remains about defining, and monitoring for, progressive VF loss.

As further glaucoma medications are developed, including therapies for potential optic nerve/GC regeneration, the need for specific paediatric studies will increase especially as authorities such as the European Medicines Agency require specific paediatric investigation plans. This will increase the need for reliable study endpoints appropriate for childhood glaucoma. Thus, further research is required to provide robust evidence in areas where divergence of opinions remain, repeating a consensus process if necessary once these data are available. In particular, there is a paucity of prospective, longitudinal research investigating optimal methods for detecting progressive VF loss in children with glaucoma, which is an area that has been investigated extensively in adults [20–22] where it is the prime measure of glaucoma stability versus progression.

The evidence of consensus reported here and the recommendations that emanate from this should aid structured decisions by clinicians, informed by knowledge of their individual patients, regarding the use of perimetry in the routine management of children with glaucoma.

## Summary

### What was known before

- Assessment of visual fields provides key information for management decisions in adult glaucoma.
- Limited information was available about utilising perimetry in childhood glaucoma, from the age at which testing should be started, to test selection and interpreting findings.

### What this study adds

- Expert consensus recommends starting static perimetry at approximately 7 years of age, adding a kinetic component approximately 1 year later. Test quality should be assessed both qualitatively and using quantitative measures. Static test selection should be geared towards shorter algorithms.
- Areas for research priority include longitudinal studies to investigate optimal use of perimetry in detecting progressive visual field loss in children with glaucoma.

## Supplementary information

Round 1 Questionnaire

Round 2 Questionnaire
