# Supplementary material for: Study of Optimal Perimetric Testing In Children (OPTIC): developing consensus and setting research priorities for perimetry in the management of children with glaucoma
Source: Eye (Lond). 2021 Jun 21;36(6):1281–7. doi: 10.1038/s41433-021-01584-0 (PMC9151738; doi:10.1038/s41433-021-01584-0)
Supplement: Supplementary file 1 — Round 1 Questionnaire [file 41433_2021_1584_MOESM1_ESM.pdf]

Assessment of visual fields in children with glaucoma: Building a consensus statement

The questionnaire is divided into 5 domains; (A) Starting perimetry in childhood, (B) Assessing perimetric test quality, (C) Test selection, (D) Assessing for progressive visual field loss and (E) Use of perimetry in routine clinical practice.

There are three types of questions:

1. Questions asking you to enter a specific value
2. Questions that ask how much you agree/disagree with a statement
3. Questions that ask you to mark all responses that apply

There is space for any additional comments you may have at the end.

Table of Contents

A. Starting perimetry in childhood....3
B. Assessing perimetric test quality....3
Using automated indices derived during static perimetry....3
Using other indices for static/kinetic perimetry....4
C. Test selection....4
Static perimetry....4
....5
Kinetic perimetry....5
Combined static and kinetic perimetry....5
D. Assessing for progressive visual field loss....5
E. Use of perimetry in routine clinical practice....6
F. Comments....7

A. Starting perimetry in childhood

1. What do you think is the minimum age at which perimetry should routinely be attempted in children with glaucoma?

☐ years

B. Assessing perimetric test quality

Using automated indices derived during static perimetry

2. Assessing percentage/frequency of false positives is useful for assessing perimetric test quality.

☐ Strongly agree ☐ Agree ☐ Neither agree nor disagree ☐ Disagree ☐ Strongly disagree

a. The threshold for grading a test as poor quality should be a false positive score greater than:

☐ 5% ☐ 10% ☐ 15% ☐ 20% ☐ 25% ☐ 30% ☐ 35% ☐ 40% ☐ 50%

☐ Other (specify)

3. Assessing percentage/frequency of false negatives is useful for assessing perimetric test quality.

☐ Strongly agree ☐ Agree ☐ Neither agree nor disagree ☐ Disagree ☐ Strongly disagree

b. The threshold for grading a test as poor quality should be a false negative score greater than:

☐ 5% ☐ 10% ☐ 15% ☐ 20% ☐ 25% ☐ 30% ☐ 35% ☐ 40% ☐ 50%

☐ Other (specify)

4. Assessing percentage/frequency of fixation losses is useful for assessing perimetric test quality.

☐ Strongly agree ☐ Agree ☐ Neither agree nor disagree ☐ Disagree ☐ Strongly disagree

c. The threshold for grading a test as poor quality should be a fixation loss score greater than:

☐ 5% ☐ 10% ☐ 15% ☐ 20% ☐ 25% ☐ 30% ☐ 35% ☐ 40% ☐ 50%

☐ Other (specify)

Using other indices for static/kinetic perimetry

5. Assessing patient behaviour qualitatively (documenting co-operation, response to stimuli, fixation, and behaviour etc.) is useful for assessing perimetric test quality.

☐ Strongly agree ☐ Agree ☐ Neither agree nor disagree ☐ Disagree ☐ Strongly disagree

C. Test selection

Static perimetry

As larger VF test areas are more informative but take longer to assess, the following questions are designed to understand the optimal balance for testing children.

6. What test area (in deg^2) gives the most information about glaucomatous field loss?

☐ 10 ☐ 24 ☐ 30 ☐ Other (specify)

7. What test area (in deg^2) should be routinely assessed in children?

☐ 10 ☐ 24 30 Other (specify)

If the two areas above (questions 6 & 7) are different, please specify reasons for your choices.

8. In children, shorter algorithms (e.g. SITA FAST and G-TOP) are preferable to their longer counterparts (SITA standard and G).

☐ Strongly agree ☐ Agree ☐ Neither agree nor disagree ☐ Disagree ☐ Strongly disagree

Please state a reason for this response:

Kinetic perimetry

Kinetic perimetry is not commonly performed routinely in children with glaucoma.

9. What factors would make you consider assessment of VF extent using kinetic perimetry?

   a. ☐ I would perform kinetic perimetry routinely in any child with glaucoma

   b. ☐ Presence of mild VF loss

   c. ☐ Presence of moderate VF loss

   d. ☐ Presence of severe VF loss

   e. ☐ I would not consider performing kinetic perimetry

Combined static and kinetic perimetry

10. Combining static perimetry and assessment of the far-peripheral field using kinetic perimetry is useful in assessing visual fields in children with glaucoma.

☐ Strongly agree ☐ Agree ☐ Neither agree nor disagree ☐ Disagree ☐ Strongly disagree

11. What is the age at which combined perimetry can be used successfully in children?

    ☐ years

D. Assessing for progressive visual field loss

12. Please define the thresholds of the following parameters that you consider evidence of progressive visual field loss:

    Loss of ☐ dB mean deviation (MD)

    using data from at least ☐ consecutive tests

13. Fellow eyes in unilateral glaucoma can serve as 'controls' within individual children, aiding monitoring of visual field progression.

☐ Strongly agree ☐ Agree ☐ Neither agree nor disagree ☐ Disagree ☐ Strongly disagree

E. Use of perimetry in routine clinical practice

14. How often should routine perimetry be undertaken in children?

☐ 3 months ☐ 6 months ☐ 9 months ☐ 12 months

☐ Other (specify)

15. Ideally, children should be assessed with the same perimeter/algorithm throughout childhood.

☐ Strongly agree ☐ Agree ☐ Neither agree nor disagree ☐ Disagree ☐ Strongly disagree

16. Longer algorithms offer greater precision in detecting progressive visual field loss.

☐ Strongly agree ☐ Agree ☐ Neither agree nor disagree ☐ Disagree ☐ Strongly disagree

17. If using shorter algorithms early in childhood (e.g. SITA FAST and G-TOP), children/young people should be switched to longer algorithms when appropriate (SITA standard and G).

☐ Strongly agree ☐ Agree ☐ Neither agree nor disagree ☐ Disagree ☐ Strongly disagree

Thank you for taking the time to complete this questionnaire. Please return via email to dipesh_patel@ucl.ac.uk.

F. Comments
