# Supplementary material for: Study of Optimal Perimetric Testing In Children (OPTIC): developing consensus and setting research priorities for perimetry in the management of children with glaucoma
Source: Eye (Lond). 2021 Jun 21;36(6):1281–7. doi: 10.1038/s41433-021-01584-0 (PMC9151738; doi:10.1038/s41433-021-01584-0)
Supplement: Supplementary file 2 — Round 2 Questionnaire [file 41433_2021_1584_MOESM2_ESM.pdf]

Assessment of visual fields in children with glaucoma – 2nd round consensus questionnaire

Thank you for completing the first round of the questionnaire.

The following questionnaire seeks to achieve a consensus opinion based on the results from the first round and follows the same format.

For values where discrepancies exist, we have taken an interquartile range (IQR, central 50% of responses) and ask you to rate your agreement with this range. We will present the IQR in the final consensus statement to demonstrate diversity of opinions. For responses with more than 5/7 in agreement (marked in green font), we have listed the statement as it will appear in the final consensus statement.

There is space for any additional comments you may have at the end.

Table of Contents

A. Starting perimetry in childhood........3
B. Assessing perimetric test quality........3
Using automated indices derived during static perimetry........3
Using other indices for static/kinetic perimetry........3
C. Test selection........4
Static perimetry........4
Kinetic perimetry........4
Combined static and kinetic perimetry........5
D. Assessing for progressive visual field loss........5
E. Use of perimetry in routine clinical practice........5
F. Comments........7

A. Starting perimetry in childhood

1. Perimetry should be started from approximately 7 years of age (IQR: 6.75-7.25).

☐ Strongly agree ☐ Agree | Neither agree nor disagree ☐ Disagree☐ Strongly disagree

B. Assessing perimetric test quality

Using automated indices derived during static perimetry

2. **False positives are a useful measure of test quality.** (7/7 in agreement)

a. False positive values over 15% (IQR: 12.5-20) indicate a test of poor quality.

☐ Strongly agree ☐ Agree ☐ Neither agree nor disagree ☐ Disagree ☐ Strongly disagree

3. **False negatives are a useful measure of test quality.** (6/7 in agreement)

a. False negative values over 20% (IQR: 12.5-22.5) indicate a test of poor quality.

☐ Strongly agree ☐ Agree ☐ Neither agree nor disagree ☐ Disagree ☐ Strongly disagree

4. **Fixation losses are a useful measure of test quality.** (6/7 in agreement)

a. Fixation losses are susceptible to artefact (such as head movement and incorrect initial plotting of the blind spot). (comment from 1/7)

☐ Strongly agree ☐ Agree ☐ Neither agree nor disagree ☐ Disagree ☐ Strongly disagree

Using other indices for static/kinetic perimetry

5. **Assessing patient behaviour qualitatively (documenting co-operation, response to stimuli, fixation, and behaviour etc.) is useful for assessing perimetric test quality.** (7/7 in agreement)

a. Qualitative (examiner) comments about test quality should always be used in adjunct to quantitative measures. (comment from 1/7)

☐ Strongly agree ☐ Agree ☐ Neither agree nor disagree ☐ Disagree ☐ Strongly disagree

C. Test selection

Static perimetry

6. **In children, due to poor concentration** (comments from 4/7)**, shorter algorithms are preferable to their longer counterparts.** (7/7 in agreement)

a. Shorter algorithms are useful to train children before undertaking longer algorithms. (comments from 2/7)

☐ Strongly agree ☐ Agree ☐ Neither agree nor disagree ☐ Disagree ☐ Strongly disagree

7. Assessing either a 30 (3/7) or 24 degree (3/7) test area is recommended for children with glaucoma.

☐ Strongly agree ☐ Agree ☐ Neither agree nor disagree ☐ Disagree ☐ Strongly disagree

8. Selecting a smaller test area (24 degrees) can offer a compromise of ease, practicality, tiring patient and information. (comment from 1/7)

☐ Strongly agree ☐ Agree ☐ Neither agree nor disagree ☐ Disagree ☐ Strongly disagree

Kinetic perimetry

9. The presence of moderate/severe VF loss is an indication to quantify VF extent using kinetic perimetry (4/7 in agreement with 1 recommending assessment routinely)

☐ Strongly agree ☐ Agree ☐ Neither agree nor disagree ☐ Disagree ☐ Strongly disagree

a. Kinetic perimetry can be a useful adjunct to static testing in those with co-operation too poor for short static testing. (comments from 2/7)

☐ Strongly agree ☐ Agree ☐ Neither agree nor disagree ☐ Disagree ☐ Strongly disagree

Combined static and kinetic perimetry

10. **Combining static perimetry and assessment of the far-peripheral field using kinetic perimetry is useful in assessing visual fields in children with glaucoma.** (5/7 in agreement)

11. Combined static and kinetic perimetry can be started in children from 7.75 (IQR: 7.5-9.5) years of age.

☐ Strongly agree ☐ Agree ☐ Neither agree nor disagree ☐ Disagree ☐ Strongly disagree

D. Assessing for progressive visual field loss

12. Evidence of VF progression can be defined as: Loss of 2 dB (IQR: 2-2.375) mean deviation (MD) using data from at least 3 (IQR: 2.5-3) consecutive tests.

☐ Strongly agree ☐ Agree ☐ Neither agree nor disagree ☐ Disagree ☐ Strongly disagree

E. Use of perimetry in routine clinical practice

13. **Fellow eyes in unilateral glaucoma can serve as 'controls' within individual children, aiding monitoring of visual field progression.** (5/7 in agreement)

14. Perimetry in children should be undertaken routinely every 7.5 months (IQR: 6-11.25).

☐ Strongly agree ☐ Agree ☐ Neither agree nor disagree ☐ Disagree ☐ Strongly disagree

a. More frequent testing is warranted if there is suspicion of VF deterioration or poor IOP control. (comments from 3/7)

☐ Strongly agree ☐ Agree ☐ Neither agree nor disagree ☐ Disagree ☐ Strongly disagree

15. **Currently, there exists a divergence of opinions with regards to the use of perimetry in the long-term management of childhood glaucoma.**

16. **There is disagreement about whether longer algorithms offer greater precision in detecting progressive VF loss.**

17. **There is disagreement about whether to switch to longer algorithms later in childhood.**

18. **Thus, it is recommended to use the same perimetric strategy throughout childhood.** (5/7 in agreement)

Thank you for taking the time to complete this questionnaire. Please return via email to dipesh_patel@ucl.ac.uk.

F. Comments
